# Consistency of Published Results on the Pathogen *Batrachochytrium dendrobatidis* in Madagascar: Formal Comment on Kolby et al. Rapid Response to Evaluate the Presence of Amphibian Chytrid Fungus (*Batrachochytrium dendrobatidis*) and Ranavirus in Wild Amphibian Populations in Madagascar

**DOI:** 10.1371/journal.pone.0135900

**Published:** 2015-10-14

**Authors:** Molly C. Bletz, Gonçalo M. Rosa, Franco Andreone, Elodie A. Courtois, Dirk S. Schmeller, Nirhy H. C. Rabibisoa, Falitiana C. E. Rabemananjara, Liliane Raharivololoniaina, Miguel Vences, Ché Weldon, Devin Edmonds, Christopher J. Raxworthy, Reid N. Harris, Matthew C. Fisher, Angelica Crottini

**Affiliations:** 1 Technische Universitat Braunschweig, Division of Evolutionary Biology, Zoological Institute, Mendelssohnstr. 4, 38106, Braunschweig, Germany; 2 Durrell Institute of Conservation and Ecology, School of Anthropology and Conservation, University of Kent, Canterbury, Kent, CT2 7NR, United Kingdom; 3 Institute of Zoology, Zoological Society of London, Regent’s Park, London, NW1 4RY, United Kingdom; 4 Centro de Biologia Ambiental, Faculdade de Ciências da Universidade de Lisboa, Bloco 2, Piso 5, Campo Grande, 1749–016, Lisbon, Portugal; 5 Museo Regionale di Scienze Naturali, Via G. Giolitti, 36, I-10123, Torino, Italy; 6 IUCN SSC Amphibian Specialist Group-Madagascar, Antananarivo, Madagascar; 7 CNRS-Guyane, USR 3456, 2 avenue Gustave Charlery, 97300, Cayenne, Guyane Française; 8 Station d’écologie expérimentale du CNRS à Moulis, USR 2936, 2 route du CNRS, 09200, Moulis, France; 9 UFZ–Helmholtz Centre for Environmental Research, Department of Conservation Biology, Permoserstr. 15, 04318, Leipzig, Germany; 10 EcoLab (Laboratoire Ecologie Fonctionnelle et Environement), CNRS/Université de Toulouse, UPS, INPT, 118 route de Narbonne, 31062, Toulouse, France; 11 Département de Biologie Animale et Ecologie, Faculté des Sciences, University of Mahajanga, Ambondrona, B.P. 652, Mahajanga 401, Madagascar; 12 University of Antananarivo, BP 566, Antananarivo 101, Antananarivo, Madagascar; 13 Unit for Environmental Sciences and Management, North-West University, Private Bag X6001, Potchefstroom, 2520, South Africa; 14 Association Mitsinjo, Lot 104 A Andasibe Gare, Andasibe, 514, Madagascar; 15 Department of Herpetology, American Museum of Natural History, Central Park West at 79^th^ St., New York, NY, 10024, United States of America; 16 Department of Biology, James Madison University, Harrisonburg, VA, 22807, United States of America; 17 Department of Infectious Disease Epidemiology, Imperial College London, London, W2 1PG, United Kingdom; 18 CIBIO Research Centre in Biodiversity and Genetic Resources, InBIO, Universidade do Porto, Campus Agrário de Vairão, Rua Padre Armando Quintas, N°7, 4485–661 Vairão, Vila do Conde, Portugal; University of South Dakota, UNITED STATES

A recent paper by Kolby et al. [1], surveying for *Batrachochytrium dendrobatidis (Bd)* and ranavirus in Madagascar, presents results for 508 amphibian specimens and 68 water bodies sampled during a 2-month period of the 2013–14 wet season. Kolby et al. [1] did not detect *Bd* in any of the samples, presenting evidence that add to our understanding of *Bd* dynamics in Madagascar. Earlier in 2015, we published “Widespread presence of the pathogenic fungus *Batrachochytrium dendrobatidis* in wild amphibian communities in Madagascar” in the journal Scientific Reports [2]. We presented rigorous spatial and temporal surveillance data for 4,155 amphibians sampled across a 10-year period, and used two independent molecular diagnostics to demonstrate the occurrence of a molecular signature of *Bd* infection at multiple locations across the island. We focus here on solely the *Bd* results, which directly relate to our published study.

While the conclusions of *Bd’s* occurrence and prevalence in Madagascar may appear to conflict between these papers, upon closer investigation the data sets actually complement each other. Our evidence for *Bd’s* presence and its widespread incidence is based on multi-year monitoring data carried out through the National Monitoring Program [3] and allied survey efforts, occurring in both the wet and dry season. Our data collected during the same time as Kolby et al.’s sampling (2013–14 wet season), is consistent with their recently published results [1,2] (summarized in Table 1). Therefore, Kolby et al.’s conclusion that our data “highly contradict” those reported in their study is inaccurate. In the 2013–14 wet season, we sampled 569 frogs from 8 locations, of which only 3 samples showed a positive signal for *Bd*. The positive samples were collected from one individual at each of three sites: Antoetra, Ranomafana, and Ankaratra. While both datasets (the sampling reported in Kolby et al. 2015 and the wet season 2013–2014 sampling reported in Bletz et al. 2015) surveyed numerous individuals and locations across the island, there are some differences in sampling locations. More specifically, one of our positive occurrences came from Antoetra, which was not surveyed by Kolby et al [1]. Two of our positives do come from locations surveyed by both groups: Ranomafana and Ankaratra; if we combine the sub-sites within these locations, the prevalence is 0.0043 and 0.0062 respectively, which falls within the prevalence confidence intervals presented in Kolby et al. [1] (Table 1). This same logic is used by Kolby et al. [1] to show the complementarity of their field survey data and Kolby’s previous work showing *Bd*’s presence in amphibians imported into the US from Madagascar [4]. Both datasets are consistent with the conclusion that *Bd* had a very low prevalence during the 2013–2014 wet season.

**Table 1:** Summary of published *Bd* survey data for the 2013–2014 wet season.

	Bletz et al. 2015 [2]				Kolby et al. 2015 [1]			
Location	Year-Month	Detection (# positive)	Sample Size	Prevalence	Year-Month	Detection (# positive)	Sample Size	Prevalence CI
**Ambohitantely**	2014-Jan	NEG	30		—	—	—	
**An'Ala**	2014-Feb	NEG	31		—	—	—	
**Andasibe**	2014-Feb	NEG	15		2014-Feb-Apr	NEG	33	0-.104
**Andringitra**	—	—	—		2014-Feb-Apr	NEG	90	0–0.041
**Ankarafantsika**	—	—	—		2014-Feb-Apr	NEG	55	0–0.065
**Ankaratra**	2013-Dec	POS(1)	161	0.006	2014-Feb-Apr	NEG	67	0–0.054
**Antananarivo**	—	—	—		2014-Feb-Apr	NEG	35	0–0.099
**Antoetra**	2014-Jan	POS(1)	36	0.028	—	—	—	
**Fierenana**	2014-Jan	NEG	29		—	—	—	
**Isalo**	—	—	—		2014-Feb-Apr	NEG	46	0–0.077
**Ranomafana**	2014-Jan	POS (1)	231	0.004	2014-Feb-Apr	NEG	109	0–0.034
**Toamasina**	—	—	—		2014-Feb-Apr	NEG	9	0–0.299
**Torotorofotsy**	2014-Feb	NEG	36		—	—	—	
**Zahamena**	—	—	—		2014-Feb-Apr	NEG	64	0–0.057

Table 1. Published data collected during the wet season of 2013/2014 (Dec 2013-March 2014) by Bletz et al. [2] and Kolby et al. [1] summarized by major locations. “—”Indicates when data were not collected.

The low prevalence detected in the wet season may likely be explained by seasonality of *Bd*. Both papers discuss the possibility of seasonal patterns, where *Bd* prevalence decreases in the warmer, wetter season and increases in the cooler, dryer season due to climatic or so-far un-described environmental factors. This phenomenon is not unusual and several studies have noted a high degree of seasonal variation in the prevalence of *Bd* (e.g. [5, 6, 7, 8]). In our published study [2] we present preliminary evidence of a seasonal pattern of *Bd*, showing that prevalence and/or detection was greater in the dryer, cooler season (May-Oct) than the wetter, warmer season (Nov-April) [2]. This seasonal pattern we documented likely explains the lack of detection by Kolby et al. since they sampled only in the wet (and warmer) season. Kolby et al. [1] supplement their individual sampling with the analysis of filtered water from natural habitats. In this case, the lack of *Bd* detection might be associated with increased water flow due to increased rainfall during the wet season, which could lower the concentration of *Bd* zoospores to undetectable levels. Additionally, water-filters of natural water bodies have also been found to be less sensitive than direct sampling of amphibians [9,10]. To better understand seasonal variation as well as other factors such as geographic distribution and host species variation, additional sampling across wet and dry season in a uniform and standardize manner will be important.

The major difference between these papers is that we draw from data collected in multiple years and seasons and from a much larger sample of amphibians with further validation using chytrid lineage-based PCR amplification, making our study more comprehensive in nature. Kolby et al. [1] surveyed for a 2-month period, which makes it difficult to make general assumptions about pathogen occurrence from such a small snap shot in time. Our data set thus allows for the conclusion of the “widespread presence of *Bd*” as we document repeated detections of *Bd* at geographically distant locations in Madagascar albeit with varying degree of prevalence among sites and seasons. We also have secondary confirmation and validation of *Bd’s* presence from an independent non-nuclear lineage specific qPCR designed to the *Bd* mtDNA locus, which is unique to our study. Kolby et al. [1] suggest that *Bd* in Madagascar cannot yet be described with certainty in part due to the variability of sampling and detection methods. We acknowledge ourselves that our use of various methods may confound some of our findings, such as the seasonal pattern of *Bd*; however, and importantly, this does not negate or question the evidence for *Bd*-positive samples collected from Madagascar.

While the data presented by both studies indicate a low prevalence of *Bd* in Madagascar in 2014, we argue that the additional multi-year data we have collected strongly supports the occurrence of one or more *Bd* lineage(s) in the samples collected from wild Madagascar amphibians. A similar conclusion was also made by Kolby et al. [4] based on their observations of *Bd* in wild-caught frogs from Madagascar that were imported into the USA. Definitive and final confirmation of *Bd* in Madagascar awaits histopathology, isolation of a *Bd* culture, and/or genome sequencing. These additional analyses can clarify whether Madagascar is facing the panzootic, hypervirulent *Bd-*GPL or a different (possibly endemic) *Bd*-lineage.

More importantly, our results may have serious conservation implications. We presented strong evidence that at least one lineage of *Bd* exists in Madagascar, with increased prevalence at some locations during the dry season. It remains to be understood if this genotype is virulent with respect to the resident anuran fauna and capable of causing population declines. Using the ‘precautionary principle’ in reacting to suspected introductions of novel emerging infectious diseases infecting wildlife [11] it is essential to initiate conservation actions. Continuing ongoing population monitoring of Madagascar's amphibians and pathogen surveillance through the NMP are therefore essential and are a priority of the national amphibian conservation strategy for the country known as ‘A Conservation Strategy for the Amphibians of Madagascar (ACSAM) [12,13]. If it is relatively hypovirulent, it gives conservationists time to engage in mitigation strategies and to plan for the possible (and likely inevitable) arrival of a virulent genotype, which could threaten the diverse, endemic frog communities.
